# Chronic koro-like symptoms – two case reports

**DOI:** 10.1186/1471-244X-5-34

**Published:** 2005-10-12

**Authors:** Nilamadhab Kar

**Affiliations:** 1 Wolverhampton Primary Care Trust, Corner House Resource Centre, 300, Dunstall Road, Wolverhampton, WV6 0NZ, United Kingdom

## Abstract

**Background:**

Koro is a culture bound syndrome, which has been reported usually from Asian countries. It has been described as an acute, brief lasting illness, which often occurs in epidemics. There is no description in literature of a chronic form of this syndrome.

**Case presentation:**

Two sporadic cases with koro-like symptoms from East India are presented where the illness had a chronic course with durations spanning more than ten years. In contrast to acute, good prognosis, psycho-education responsive form that is usually seen in epidemics; the chronic form, appeared to be associated with greater morbidity and poorer response to interventions.

**Conclusion:**

There is a possibility of a chronic form of koro syndrome.

## Background

Koro is considered to be a cultural-bound syndrome, characterised by the belief of retraction of the genitals into the abdomen and is associated with anxiety symptoms [1-3]. It has been commonly reported from India and other Southern Asian countries. It often manifests as epidemics [4,5] though sporadic cases are also noted [6]. The syndrome is described to be short lasting. There is no description in literature of a chronic form of this syndrome. Two patients with koro-like symptoms from Orissa in East India are presented here, whose symptoms continued for prolonged duration of more than ten years, highlighting the possibility of a chronic form of this syndrome.

## Case presentation

### Case: 1

A 30-year-old unmarried, male, clerk presented with the persistent fear that his penis was shrinking gradually for last 12 years. He stated that due to the shrinkage he remained weak, anxious and sleepless. He continuously brooded over it. He got panicky whenever he saw his penis, as he felt that it is shrinking back, and remained perturbed for days together after that. Due to this he consciously avoided to see or contact his penis, even in bathroom. He explained that it was due to his nocturnal emissions and a couple of masturbatory acts in middle adolescence. He was extremely religious and attended religious institutions (like that of *Satsang *and *Brahmakumaris*). He considered penis as sacred and its gradual shrinkage as an omen of his ultimate destruction and death. He believed that this was due to sinful (anti-religious) incidences of nocturnal emission and masturbatory acts. He had decided not to marry in order to avoid any possible retraction. He remained preoccupied with these thoughts, avoided company, and interaction with females. He requested for medicines that would stop further retraction.

He had been given anxiolytics, sedatives, antidepressants and anti-psychotics at various point of time by many practitioners and had been counselled. His symptoms persisted with intermittent exacerbations without much relief.

Unlike obsession his thoughts were never resisted. His belief had influenced his behaviour and way of life, was shakeable, and appeared more like overvalued ideas. His mood state was predominantly anxious with occasional panic attacks associated with somatic equivalents. He did not have any other major psychiatric syndrome.

### Case: 2

A 41-year-old unmarried, unemployed male from a business family, presented with the complaints of gradual retraction of penis and scrotum into the abdomen. He had frequent panic attacks feeling that the end had come. The symptoms had persisted more than 15 years with a waxing and waning course. During exacerbations he spent most of his time measuring the penis by a scale and pulling it in order to bring it out of abdomen. He tied a string around it and attached it to a hook above to prevent its shrinkage during night. There was a history of excessive sexual indulgence in the early twenties with prostitutes and he had suffered repeatedly from sexually transmitted diseases characterised by discharges (probably gonorrhoea) and ulcers (probably syphilis). He was adequately treated for that. He did not have remorse or guilt over those happenings. He continued visiting prostitutes occasionally and described no inadequacy in sex. He engaged his partner in fellatio in order to bring the penis out. He refused marriage, as he feared that his life was at stake due to this shrinkage as he had had many experiences of nearing death due to that. He did not have regular work and was mostly dependent on the family.

He had been treated with anxiolytics and occasionally with antipsychotic medications with minimal benefit. He had received psycho-education. He frequently engaged in doctor shopping and tried to see doctors before the appointment day expressing concern over his symptoms. He often used medicines from other fields of medicine (homeopathy, Ayurveda, unani) to prevent the retraction.

## Conclusion

The presenting symptoms of these two patients, characterised by excessive anxiety and belief of shrinkage of genitalia, are similar to those found in koro patients. Age of onset of the koro symptoms in the index patients are comparable to that (20–40 years) observed in the epidemics [5]. Factors such as extramarital intercourse, venereal disease and scrotal filaria were found to be significantly commoner in koro patients [7]. One of the index patients had veneral diseases and had contact with prostitutes. Significant premorbid or sexual psychopathologies were absent in most cases in epidemics [5], so also in the index cases. The symptoms of these patients were not secondary to any other major psychiatric disorder.

The findings in these index cases are supported by the view that individual koro patients exhibit a symptomatology indicative of major psychiatric conditions (i.e. psychosis or affective disorder), and appear unrelated to collective episodes which involve social, cultural, cognitive and physiological factors in the diffusion of koro-related beliefs [6]. A sporadic case of koro was reported to be associated with psychotic depression [8].

However compared to koro patients reported in literature, the index cases had few differences considering duration of illness, continued presence of significant psychiatric morbidity characterised by koro-like symptoms which affected their life significantly, and non-response to interventions. Multiple episodes have been described in some patients in koro epidemics but with only minor residual symptoms [5], however in the index patients the symptoms have been chronic. Majority of the individuals affected in koro epidemic in India were reported from the lower socio-economic strata and were poorly educated, which were not the case with the index patients. Epidemics of koro were known to be contained or benefited by mass education programmes [9]. In the index patients the symptoms of koro did not respond in spite of various interventions, which included individual sessions of psycho-education.

While the issues concerning phenomenology, diagnosis and nosology of koro are still being discussed [5,6,10], it is apparent that koro, which presents both sporadically and in epidemics as an acute anxiety state, may also have a chronic form. In contrast to acute, good prognosis, psycho-education responsive form that is usually seen in epidemics; the chronic form, appears to be sporadic with greater morbidity, and with poorer response to interventions.

The underlying dynamics resulting in chronicity of symptoms of koro are not clear. It is possible, as observed before [11], that many psychiatric symptoms that share a similar 'surface grammar' differ in their 'deep grammar' or structure. Though the index cases suggest that koro can be chronic, with a poorer prognosis; further information regarding this presentation and factors behind it are needed.

## Competing interests

The author(s) declares that he has no competing interests.

## Authors' contributions

NK examined the patients, treated, followed up, conceptualised, did literature survey and wrote the paper.

## Pre-publication history

The pre-publication history for this paper can be accessed here:


